# The role of childhood trauma in bipolar disorders

**DOI:** 10.1186/s40345-015-0042-0

**Published:** 2016-01-13

**Authors:** Monica Aas, Chantal Henry, Ole A. Andreassen, Frank Bellivier, Ingrid Melle, Bruno Etain

**Affiliations:** NORMENT, KG Jebsen Centre for Psychosis Research, TOP Study Group, Division of Mental Health and Addiction, Institute of Clinical Medicine, University of Oslo, Bygg 49, Ullevål Sykehus, Nydalen, PO Box 4956, 0424 Oslo, Norway; Division of Mental Health and Addiction, Oslo University Hospital, Oslo, Norway; AP-HP, Hôpitaux Universitaires Henri Mondor, DHU Pepsy, Pôle de Psychiatrie, 94000 Créteil, France; Université Paris Est, Faculté de Médecine, 94000 Créteil, France; Inserm, U955, 94000 Créteil, France; Fondation Fondamental, Créteil, France; AP-HP, Hôpital Fernand Widal, Pôle Addictologie-Toxicologie-Psychiatrie and Université Paris-7, Paris, France; ENBREC, European Network of Bipolar Research Expert Centres (ENBREC), Paris, France

## Abstract

This review will discuss the role of childhood trauma in bipolar disorders. Relevant studies were identified via Medline (PubMed) and PsycINFO databases published up to and including July 2015. This review contributes to a new understanding of the negative consequences of early life stress, as well as setting childhood trauma in a biological context of susceptibility and discussing novel long-term pathophysiological consequences in bipolar disorders. Childhood traumatic events are risk factors for developing bipolar disorders, in addition to a more severe clinical presentation over time (primarily an earlier age at onset and an increased risk of suicide attempt and substance misuse). Childhood trauma leads to alterations of affect regulation, impulse control, and cognitive functioning that might decrease the ability to cope with later stressors. Childhood trauma interacts with several genes belonging to several different biological pathways [Hypothalamic–pituitary–adrenal (HPA) axis, serotonergic transmission, neuroplasticity, immunity, calcium signaling, and circadian rhythms] to decrease the age at the onset of the disorder or increase the risk of suicide. Epigenetic factors may also be involved in the neurobiological consequences of childhood trauma in bipolar disorder. Biological sequelae such as chronic inflammation, sleep disturbance, or telomere shortening are potential mediators of the negative effects of childhood trauma in bipolar disorders, in particular with regard to physical health. The main clinical implication is to systematically assess childhood trauma in patients with bipolar disorders, or at least in those with a severe or instable course. The challenge for the next years will be to fill the gap between clinical and fundamental research and routine practice, since recommendations for managing this specific population are lacking. In particular, little is known on which psychotherapies should be provided or which targets therapists should focus on, as well as how childhood trauma could explain the resistance to mood stabilizers.

## Childhood trauma as a risk factor for psychiatric disorders across diagnostic boundaries

The WHO (World Health Organization) estimates that a quarter of all adults report having been physically abused as a child, with childhood sexual abuse reported by one in five women and one in 13 men (WHO Library Cataloguing-in-Publication Data 2006). In individuals suffering from severe mental disorders, childhood trauma is reported at a much higher rate. For example, a study from the National Epidemiologic Survey on Alcohol and Related Conditions (NESARC) has investigated the risk for any DSM-IV psychiatric disorders when exposed to childhood physical abuse. Such an exposure is associated with increased risks for any substance use disorders (OR = 1.24 [1.07, 1.44]), psychotic disorders (OR = 1.27 [1.00, 1.61]), any mood disorders (OR = 1.41 [1.25, 1.60]), any anxiety disorders (OR = 1.56 [1.38, 1.77]), and any suicide attempts (OR = 1.57 [1.26, 1.96]) (Sugaya et al. 2012).

Three previous reviews of the literature concerning childhood trauma in BD have been published within a short period of time (Etain et al. 2008; Fisher and Hosang 2010; Daruy-Filho et al. 2011), all showing associations between childhood trauma and BD susceptibility and/or severity. Since a significant number of studies have been published since 2011 in this domain, an update of these reviews is particularly timely. Furthermore, issues regarding trauma as a whole, as compared with trauma subtypes (emotional, physical and sexual trauma) or gender, have emerged and will be discussed in this review. A considerable amount of literature has also been added to investigate the psychological (dimensions of psychopathology) and biological pathways (in particular, gene/environment interaction studies) leading from childhood trauma to BD susceptibility and severity. Lastly, we will illustrate that, despite the growing volume of literature in this field, gaps between clinical and biological research and therapeutic implications are still wide.

## Childhood trauma as a risk factor in BD

In 2010, Fisher identified only six robust studies exploring childhood trauma in BD suggesting an association with childhood trauma. Since then, further studies have consolidated the level of proof (Alvarez et al. 2011; Etain et al. 2010; Janiri et al. 2015; Watson et al. 2013). For example, a case-control study assessing 206 patients with BD compared to 94 controls using the Childhood Trauma Questionnaire (CTQ) (Bernstein et al. 1994) shows that multiple traumas are more frequent in patients with BD than in controls (63 *versus* 33 %). Additionally, among trauma subtypes (emotional, physical, and sexual abuses), only emotional abuse has a suggestive dose-effect with BD (Alvarez et al. 2011; Etain et al. 2010). Replication of these results using a smaller sample of 60 patients with BD *versus* 55 controls, assessed with the CTQ, shows an association between the BD and CTQ total score. All subscale scores are significantly higher in patients with BD compared to controls, apart from sexual abuse (Watson et al. 2013). Janiri et al. (2015) demonstrate that BD type I patients differ significantly from healthy controls for sexual abuse, and BD type II patients differ from healthy controls for emotional neglect. Maniglio (2013) also reviews 20 studies, including 3407 young and adult patients with BD across 10 countries and three continents, concluding that, compared to healthy individuals, patients with BD report higher rates of child sexual abuse.

Therefore, childhood trauma in all its subcomponents appears to be highly associated with BD, although the specific role of each trauma subtype (emotional, physical or sexual abuse) remains a subject of debate.

## Childhood trauma and severity of the clinical expression of BD

There are consistent indications that childhood traumatic events are associated with various severe clinical characteristics of BD, including an earlier onset of the illness (Garno et al. 2005), a rapid cycling course (Garno et al. 2005), psychotic features (Bebbington et al. 2004; Hammersley et al. 2003; Janssen et al. 2004; Shevlin et al. 2007), a higher number of lifetime mood episodes (Brown et al. 2005; Nemeroff, 2004; Weber et al. 2008), and suicide ideation and attempts (Alvarez et al. 2011). However, the quality of these studies has been reported to vary (Daruy-Filho et al. 2011), thus reducing the potential relevance of the findings. Indeed, in a review of 18 studies performed by Daruy-Filho and colleagues (2011), several limitations have been highlighted, including a lack of use of a structured clinical interview for diagnosis, a lack of use of a standardized trauma assessment, a low sample size (less than 100 patients) and insufficient measures of current mood state as a potential confound in trauma assessment (Daruy-Filho et al. 2011). These potential biases are taken into account by a recent large study of patients with BD (*n* = 587), thereby confirming childhood trauma as being associated with more severe clinical features in BD, including an earlier age of onset, an increased risk of at least one lifetime suicide attempt, rapid cycling, an increased number of mood episodes and substance misuse (Etain et al. 2013a).

Three results appear consistently replicated across studies in BD: (1) the association between childhood trauma and an earlier age at onset; (2) the increased risk for suicide attempts; and (3) comorbid substance misuse. Daruy-Filho et al. (2011) underscored this fact, and since then further replications regarding these three clinical characteristics have been published.

We have demonstrated a dose effect of trauma exposure on the age at onset of BD (Etain et al. 2013a). A recent study of 132 patients with BD from China reports associations between emotional abuse and neglect and an earlier age at onset (Li et al. 2014). Post et al. (2015) also replicate these findings, showing differences in age at onset according to the absence/presence of verbal, physical, and sexual abuses. Therefore, exposure to early life stress seems to consistently lower the threshold for developing BD.

Other replicated findings have been obtained for associations between childhood trauma, suicide attempts, and substance misuse in BD, following initial findings reviewed by Daruy-Filho et al. (2011). In a French-Norwegian sample of patients with BD, both emotional and sexual abuse are independent predictors of suicide attempts (OR = 1.60 [95 % CI 1.07–2.39] and OR = 1.80 [95 % CI 1.14–2.86], respectively) (Etain et al. 2013a). A recent review by Maniglio (2013) similarly highlights the influence of childhood sexual abuse on the course and severity of BD, including associations with suicide attempts, alcohol and⁄or drug abuse or dependence. The association between childhood trauma and suicide attempt or substance misuse is probably not specific to BD. Indeed, a 25-year-long prospective study examining the development of illicit drug use from a birth cohort of 1265 New Zealand children finds that childhood sexual and physical abuse are the main predictors for illicit drug use at age 16–25 (Fergusson et al. 2008). This is consistent with the findings obtained by the NESARC, with associations between physical abuse and an increased risk for any substance use disorders (Sugaya et al. 2012). Moreover, a meta-analysis of longitudinal studies shows an association between sexual abuse and suicide attempt with an OR = 2.43 [1.94, 3.05] (Devries et al. 2014). We can therefore assume that childhood trauma elevates the risk of suicide attempt and substance misuse in BD, this probably being independent of psychiatric diagnoses.

## Gender issues and trauma subtypes

Several studies show that females with BD report childhood trauma more frequently than males (Etain et al. 2013a; Fisher et al. 2009), with one small study showing trend levels in the same direction (Alvarez et al. 2011). For instance, Etain and colleagues (Etain et al. 2013a) indicate higher level of childhood trauma in females compared to males (CTQ total score females 43.5 ± 14.8; males 40.1 ± 12.5 *P* = 0.025). In healthy populations, childhood trauma is also more frequently reported by females than males, specifically for sexual abuse, while physical abuse is more frequently reported by healthy males (Fisher et al. 2009). Additionally, greater associations between trauma and clinical characteristics in BD are reported in females. This includes stronger associations to rapid cycling, early onset of illness, increased risk for at least one suicide attempt and more depressive episodes than in males with childhood trauma. Thus, the female gender may drive the association between childhood trauma and the clinical features of BD. This in fact is investigated in the study by Etain et al. (2013a), who find that when gender is added as a covariate in the analysis the results between childhood trauma and the clinical features of BD remain significant, thereby indicating an additive effect of gender, as well as a gender non-specific effect of childhood trauma on clinical characteristics in BD (Etain et al. 2013a).

Another important issue concerns the subtypes of childhood trauma (as compared to trauma in general) that drive these effects on clinical characteristics. Until recently, childhood physical and sexual abuses have been indicated as the strongest predictors of unfavorable clinical characteristics in BD. However, physical and sexual abuse are also the most frequently studied subtypes of childhood trauma, and only a few studies have paid any attention to emotional abuse or neglect (Daruy-Filho et al. 2011). Studies over the past few years may indicate evidence of emotional abuse as a more specific risk factor in BD (Etain et al. 2008, 2010) than other trauma subtypes. Such studies have found a higher prevalence of emotional abuse in patients with BD compared to healthy controls, even after controlling for other types of abuse. A study by Martins et al. (2014) confirms emotional abuse as a risk factor for mood disorders, including patients with BD. Moreover, patients with a history of emotional abuse have higher severity scores on all symptoms, including depression, hopelessness, suicidal ideation, anxiety, and impulsivity. These data may suggest emotional abuse as a specific risk factor for certain psychiatric disorders (possibly with anxious, depressive, and emotional core features). It should also be mentioned here that it is a challenge to separate into different subtypes of trauma, since they often occur together (for an overview of commonly investigated subtypes of childhood trauma, assessed by the CTQ please see Table 1). Future studies should clarify the specific role of each trauma subtype.

**Table 1:** Trauma subtypes assessed by the CTQ (Bernstein et al. 1994)

Trauma subtype	Definition
Emotional neglect	Failure of caretakers to meet children’s basic emotional and psychological needs, including love, belonging, nurturance, and support
Emotional abuse	Verbal assaults on a child’s sense of worth or well-being or any humiliating or demeaning behavior directed toward a child by an adult or older person
Physical neglect	Failure of caretakers to provide for a child’s basic physical needs, including food, shelter, clothing, safety, and health care
Physical abuse	Bodily assaults on a child by an adult or older person that posed a risk of- or resulted in injury
Sexual abuse	Sexual contact or conduct between a child younger than 18 years of age and an adult or older person

## Childhood trauma, psychological dimensions, cognition, and brain imaging abnormalities in BD

One might postulate that childhood trauma is driving clinical outcomes (psychiatric diagnoses or certain clinical features such as suicide attempts) through non-specific dimensions of psychopathology (e.g., affect or impulse dysregulation or abnormalities in cognitive functioning).

Affective lability is suggested as a core feature of BD (Aminoff et al. 2012; Goodman et al. 2003; Henry et al. 2008) that manifests itself as frequent and intense fluctuations in affect in response to both pleasant and unpleasant events. In BD, studies have found heightened affective lability in both manic and mixed episodes (Henry et al. 2003), as well as traits during euthymic periods (Henry et al. 2008). A previous study has linked childhood trauma (specifically emotional abuse) to later affective lability in personality disorders and in BD (Goodman et al. 2003). Also in BD, one large study (*n* = 202) and a smaller study (*n* = 42) have demonstrated that exposure to childhood trauma is associated with higher scores on affective lability, with the strongest association being that for emotional abuse (Aas et al. 2014a; Etain et al. 2008). Furthermore, in an Emotion Recognition Task, patients with BD and childhood emotional neglect have a reduced performance in recognizing anger, compared to subjects without any trauma (Russo et al. 2015). Interestingly, childhood trauma is associated with increased amygdala activation (van Harmelen et al. 2013), a brain region important for regulating fear and emotions (Aas et al. 2012; Gallagher and Chiba 1996), thus reinforcing potential links between childhood trauma, changes in emotional or affect regulation and brain imaging abnormalities (limbic system).

Childhood trauma is also a contributing factor to traits of aggression in BD (Garno et al. 2008), suggesting that childhood trauma could influence, in addition to emotional regulation, components of hostility or impulsivity that could prove to increase the risk for suicide attempt or substance misuse (Etain et al. 2013b; Parmentier et al. 2012). This could be related to the effects of childhood trauma on the brain inhibitory control network (Elton et al. 2014), and should be further investigated in BD.

BD patients also present deficits in cognitive functioning, particularly in areas of working memory, executive functioning, attention, and processing speed (Bourne et al. 2013). The mechanisms behind these impairments are poorly understood, and potentially based on both heritability and environmental susceptibility factors. Very few studies have investigated the potential role of childhood trauma on cognitive impairment in BD. A recent study of early BD demonstrates that patients with childhood trauma have poorer scores on IQ, attention, verbal memory and working memory (Bucker et al. 2013), with these associations remaining significant after taking into account current mood symptoms. Similar findings have also been reported in a more established stage of BD (Savitz et al. 2008).

The relationships between childhood trauma, dimensions of psychopathology, and cognition may help shed light on the clinical overlap or comorbidities between BD and other clinical entities such as those related to affect dysregulation, impulsivity and hostility (borderline personality, suicide behaviors and substance misuse). They could also lead to a greater vulnerability to other environmental stressors such as cannabis exposure or adulthood life events.

## Interactions between childhood trauma and later stressors in BD

A two-hits model of susceptibility has been proposed in psychosis, and could show relevance for BD (Cannon et al. 2014). Briefly, exposure to prenatal or postnatal stressors interacts with genetic factors to “prime” the brain, which leads to a vulnerability as measured by changes in brain functioning or other biomarkers. Further stressors during adolescence or young adulthood (substance misuse, stressful events) may serve to convert the vulnerability into a disorder. In BD, the interaction between childhood trauma and susceptibility genes may predispose the individual to subtle changes in certain biological and physiopathological processes of the disorder. In this context, cannabis misuse during adolescence or later life events may act in and reveal this susceptibility or increasing the rate of mood recurrences.

The additive effects of childhood trauma and cannabis misuse have been demonstrated by increased rates for rapid cycling at an earlier age at onset and suicide attempt (Aas et al. 2014b), thus supporting the hypothesis of a two-hit model of susceptibility in BD. The possible mechanism behind the co-occurrence of childhood abuse and cannabis abuse could be related to their opposite effects on the hypothalamic–pituitary–adrenal (HPA) axis (Heim et al. 2008; van Leeuwen et al. 2011). Cannabis and substance misuse have been linked to a reduction in HPA activity (van Leeuwen et al. 2011), while a history of childhood trauma has been linked to increased HPA axis activity, specifically in depression (Heim et al. 2008). As a result, it could be hypothesized that cannabis or substance use in individuals with a history of childhood trauma serve a purpose of self-medication to “regulate” the HPA axis, alongside the emotional turmoil caused by the childhood trauma events.

Several studies have examined the role of stressful adult life events and episode recurrence in BD (Cohen et al. 2004; Johnson and Roberts 1995; Swendsen et al. 1995). The study by Cohen et al. (2004), consisting of 52 patients with BD type I, finds that high levels of stressful life events in adulthood predict depressive recurrence over a one-year follow-up after controlling for clinical history and compliance. Similarly, Swendsen et al. (1995) conclude that stressful life events in adulthood are predictors of a more severe symptomatology over a one-year period. Research by Johnson and Miller (1997) observes that BD patients with severe negative life events in adulthood require a recovery period threefold longer in duration compared to those without adverse life events, as life events increase the risk of both first- and recurrent admissions in BD (Kemner et al. 2015). There is no study that specifically investigates the interaction between childhood trauma and later life events as to the severity of BD, but we can postulate that having a history of childhood trauma increases the risk for an elevated response to later negative/positive life events by increased stress reactivity (Aas et al. 2011; Aas et al. 2014c). This could be mediated by changes in affect regulation, cognitive functioning or an increased impulsivity in association with maladaptive coping mechanisms. BD patients with histories of childhood trauma may be less likely to have protective factor “buffers,” including secure attachment and social support, again enhancing a vulnerability to the negative long-term effects of childhood trauma.

## Biological systems implicated in the association between childhood trauma and BD

It is beyond the scope of this article to review all the relevant biological systems that could play a role in mediating the impact of childhood trauma on the risk of developing BD or a more severe form of the disorder. Some of them (neuroplasticity, inflammation, circadian system or HPA axis) could be much more central in BD.

A first mechanism linking childhood trauma to BD susceptibility relies on neuroplasticity mechanisms, in particular BDNF, which is a neurotrophic imperative for the growth and differentiation of neurons during brain development and the maintenance of neurons in adult life. A reduction of BDNF serum or mRNA levels (Aas et al. 2014c; Kauer-Sant’Anna et al. 2007) has been observed after exposure to traumatic events in BD. Second, independently of psychiatric diagnoses, childhood trauma induces long-term modifications in inflammation processes (Baumeister et al. 2015; Coelho et al. 2014; Tursich et al. 2014). This should be studied in patients with BD who show CRP abnormalities (Dargel et al. 2015), and various cytokines such as Interleukin (IL)-2 receptor, tumor necrosis factor-α, soluble tumor necrosis factor receptor type 1, IL-6, and IL-4 (Munkholm et al. 2013). Interestingly, some authors have proposed a potential interaction between an elevated BDNF and pro-inflammatory cytokine levels after trauma (Bucker et al. 2015), with an increase in BDNF levels being a possible attempt to neutralize the negative effects of CT on the brain.

Sleep disturbances observed in BD (Geoffroy et al. 2015; Ng et al. 2015) should also be studied in the context of childhood trauma. Indeed, in the general population, childhood adversity is a risk factor for adult sleep disorders (Baiden et al. 2015; Koskenvuo et al. 2010). Since the circadian system modulates the biological responses to stressful environmental factors (Landgraf et al. 2014), this hypothesis should require more attention. To date, only one study has explored such a hypothesis among patients with anxiety and depressive disorders and found that a high stress load in childhood was associated with alterations in several sleep parameters assessed with actigraphy (Schafer and Bader 2013).

Further research is also required regarding the impact of childhood trauma on the HPA axis functioning in BD. A meta-analysis in schizophrenia and BD shows heightened levels of morning cortisol in BD (Girshkin et al. 2014), although the determinants of such modifications and the specific place of childhood trauma among them require further investigations.

As a potential future perspective, most of these biological systems possibly interact and converge to a high physical burden and reduced life expectancy in patients with BD (Kessing et al. 2015). Indeed, a meta-analysis by Norman et al. (2012) explores the long-term consequences of childhood abuse and neglect, and finds associations not only with psychiatric, but also with physical disorders. Through long-lasting consequences on the alterations of immune-inflammatory markers, sleep parameters and HPA axis, childhood trauma might be associated with poor health conditions and, as such, this hypothesis deserves more attention.

## Underlying molecular mechanisms in BD

Several mechanisms are likely to be involved in mediating the consequences of childhood trauma at the molecular level, with interactions between childhood trauma and genetic variants, epigenetics mechanisms and shortened telomere length being only a few.

### Gene-environment interactions in BD

Exposure to childhood trauma is thought to interact with various susceptibility genes to increase the risk of BD, or to a more severe clinical expression of the expression of the disorder. This mechanism is called “gene-environment interaction,” in which the phenotypic response to an environmental factor is conditioned by the genotype of the individuals. Although the literature is scant in BD, interactions between childhood trauma and candidate genes (BNDF, 5HTTLPR, TLR2, calcium-related genes, circadian genes) have been reported to influence age at onset or suicidal risk.

Miller et al. (2013) demonstrate differences in variants of the BDNF val66met and sexual abuse on the age at onset in BD; only Met carriers exposed to childhood sexual abuse have an earlier age at onset. Benedetti et al. (2014) show an interaction between trauma and 5HTTLPR on suicidality and age at onset in BD. Here, homozygotes for the short variant of the 5HTTLPR and trauma have increased the risk of suicide attempts and an earlier age of onset compared to all other groups. Etain et al. (2015) similarly support an interaction between emotional trauma and the 5HTTLPR on the age at the onset of BD (Etain et al. 2015). Oliveira et al. (2015) demonstrate an interaction between sexual abuse and a TLR2 (Toll-Like Receptor 2 belonging to immune-inflammatory pathways) variant on the age at the onset of BD (Oliveira et al. 2015). Here, TT homozygotes who reported sexual abuse had an earlier age at the onset of BD. Anand et al. (2015) demonstrate a relationship between trauma and SNPs in or near genes coding for calcium channel activity-related proteins and an earlier age at the onset of BD. Finally, Benedetti et al. (2015) suggest a combination of specific variants of the CLOCK gene (belonging to the circadian system) in combination with early stress, which may drive the elevated prevalence of suicide attempts in BD. Here, in comparison to other groups, C carriers in the presence of a delayed sleep onset and worsening insomnia exhibit an increased risk for suicide attempts in the presence of childhood trauma.

These results suggest complex effects of trauma exposure on age at onset or suicidality in BD, moderated by genes that belong to pathways involved in neuroplasticity, inflammation, serotonergic neurotransmission, calcium signaling or circadian rhythms. Interaction between childhood trauma and genes belonging to the HPA axis pathways have already been explored for depression and psychosis, but not yet for BD (Uher 2014).

### Epigenetic molecular mechanisms

One adaptive mechanism to help modulate stress response is to act through subtle modifications of gene expression, primarily through epigenetic mechanisms such as methylation and histone modifications. Both pre- (Seckl and Meaney 2004) and perinatal (Fish et al. 2004) stress have long-term effects on the HPA axis (Herbert et al. 2006) in rodent models and humans. The altered HPA axis in adult humans exposed to childhood trauma may be related to epigenetic changes of stress regulatory genes, such as the glucocorticoid receptor (GR) gene, also called the “NR3C1 gene,” or the FKBP5 gene (Klengel et al. 2013) (a glucocorticoid receptor co-chaperone). Even if epigenetic modification in response to early environmental conditions could provide a future elegant mechanism to explain the effects of childhood adversity on an increasing risk of psychopathology in adulthood, this remains to be specifically explored in BD (Nemeroff and Binder 2014).

### Telomere length

Telomeres are the DNA-based caps and protein structures at the chromosome tips, which shorten after each cell division, their length being used as a marker of biological aging (Shalev et al. 2013), as well as being associated with diabetes mellitus type II, coronary heart disease and cancer in the general population (Haycock et al. 2014; Ma et al. 2011; Wentzensen et al. 2011; Willeit et al. 2014). A shorter telomere length may be partially related to early stressor events. Indeed, two recent review articles suggest that childhood trauma is a causal factor for the long-term reduction of telomere length (Price et al. 2013; Shalev et al. 2013). Two studies have also shown a reduced telomere length in BD compared to controls (Elvsashagen et al. 2011; Lima et al. 2014), whereas others found negative results (Colpo et al. 2015). Hence, the association between childhood trauma and telomere length, along with the association with physical health, deserve future attention in BD.

## Implications for clinical assessment and treatment

First, a routine assessment of childhood trauma in both the early phases and established cases of BD should be undertaken due to the heightened risk of developing a more severe illness over time. It has previously been reported that abuse is often inadequately assessed in psychiatric clinics (Read and Fraser 1998), an aspect to be considered in the overall assessment process. The assessment of childhood trauma is particularly required for subgroups of patients with BD with early onset, comorbidity with suicide attempts or substance misuse, high level of mood recurrences or greater mood instability (as a categorical pattern, i.e., rapid cycling or as a dimensional one, i.e., affective lability). In routine practice, an assessment should be undertaken using clinical interviews, which would also benefit from the systematic use of questionnaires. It is beyond the scope of this review to discuss in depth the respective psychometric properties of interviews and self-reports. Since more than 20 self-reports and 20 interviews exist with some difference between psychometric qualities, none of them can be recommended more than another (Roy and Perry 2004). Nevertheless, the use of the CTQ may be relevant, as it is widely used in clinical research in BD, while also exploring different subtypes of trauma and not only physical or sexual abuse.

Second, addressing childhood trauma could be a focus of early intervention strategies and approaches to potentially prevent these individuals from developing an unalterable chronic illness over time; however this is only speculation. As described in Thompson et al. (2014), examples of treatment targets (working directly on the dissociative experiences in response to trauma) could be psychological techniques, coping strategies, body awareness/mindfulness techniques and stress management. Because a history of childhood trauma is also associated with reduced functioning in childhood, reaching these individuals before illness onset could potentially reduce the severity and (hypothetically) the development of the illness. Although existing data support several psychological interventions in effectively preventing or treating the negative consequences of childhood trauma in general (eye moment desensitization and reprocessing [EMDR], trauma focused Cognitive behavioral therapy [CBT] for sexually abused children) (Ehring et al. 2014; Macdonald et al. 2012), studies in established cases of BD are sparse. Landin-Romero et al. (2013) and Mueser et al. (2008) assessed a sample of 108 patients with PTSD and either major mood disorder (85 %), schizoaffective disorder or schizophrenia (15 %), of whom 25 % also had a borderline personality disorder using CBT. CBT patients improved significantly more than patients in the treatment as usual group at a blinded post-treatment and the 3- and 6-month follow-up. Due to findings of childhood trauma being associated with affective dysregulation in BD (Aas et al. 2014a) and impulsivity (Etain et al. 2013b), as well as rapid cycling (Etain et al. 2013a), psychosocial therapies should target not only traumatic experiences per se, but also cognitive defects or emotional dysregulation linked to traumatic experiences. In this context, therapies that target emotional regulation or cognitive functioning might help counterbalance the negative effects of trauma. To date, this is under-researched in the field, but deserves some attention. This will also open discussions about how those patients with BD exposed to trauma would respond in a similar manner to “classical” or more targeted psychosocial therapies.

Studies exploring treatment resistance in BD should also integrate childhood trauma as a potential predictor of non-response. Indeed, childhood trauma has been associated with a poorer response in resistant-depression (Douglas and Porter 2012; Shamseddeen et al. 2011), obsessive–compulsive disorder and schizophrenia (Hassan and de Luca 2015; Semiz et al. 2014), but never investigated in BD. Randomized controlled trials of the efficacy of interventions could easily assess childhood trauma at baseline to study how this factor is relevant in selecting those who might or might not respond to treatment. A promising area for investigation could be a non-response to lithium, for which robust predictors are still lacking.

## Perspectives and research gaps

Childhood traumatic events are potential risk factors, both for developing BD and presenting a more severe disorder over time. This association is probably not specific to BD, with childhood trauma predisposing to transnosographic indicators of severity, such as suicide attempts or substance misuse. At a dimensional level, childhood trauma may lead to abnormalities in affect regulation, impulse control and cognitive functioning that could reduce the ability to cope with later environmental risk factors (cannabis misuse, stressful life events). This leads to the absolute recommendation that clinicians should assess childhood trauma, ideally for every patient with BD, but at least for those with a more severe, unstable or relapsing course.

Several biological pathways (including neuroplasticity, inflammation, circadian systems, and HPA axis) are likely to play a role in the long-lasting consequences of childhood trauma on the risk for (severe) BD, in addition to the high level of somatic comorbidities. Preliminary results demonstrate that candidate genes belonging to these pathways help moderate the effects of childhood trauma on age at onset and suicidality in BD. Although promising, this research is still emerging in BD, and further studies are needed to clarify the involved biological pathways. In particular, some results remain to be consolidated such as the impact on brain structures and functioning, or the identification of epigenetic or transcriptomic biomarkers, in those who are at-risk of negative consequences when exposed to trauma (versus those who would be resilient). To date, the literature has primarily focused on psychological or cognitive outcomes after childhood trauma in BD, and much attention is required for the consequences on physical health in patients with BD.

At last, a large gap is present between clinical observation on the role of childhood trauma in BD, and the quasi-absence of treatment guidelines for this population. Psychosocial interventions and their preferential targets are not yet, and the implication of childhood trauma in treatment resistance (psychotherapies or medications) is totally absent in the literature. Clinicians and researchers should therefore gather to foster this reflection that would contribute to more personalized care plans for those exposed to trauma. Given the importance of trauma in prognosis and course in patients with BD, we should not only content ourselves with simply extrapolating the PTSD literature in terms of management, but should also provide evidence-based trauma interventions in BD.
